# Metabolic and bariatric surgery in patients with class I obesity; a two-year follow-up

**DOI:** 10.1186/s12893-023-02295-x

**Published:** 2024-01-03

**Authors:** Mohammad Kermansaravi, Rohollah Valizadeh, Masoumeh Shahsavan, Seyyed Adel Maleknia, Foolad Eghbali, Abdolreza Pazouki, Shahab Shahabi Shahmiri

**Affiliations:** 1 Department of Surgery, Minimally Invasive Surgery Research Center, Division of Minimally Invasive and Bariatric Surgery, Rasool-e Akram Hospital, School of Medicine, Iran University of Medical Sciences, Tehran, Iran; 2 Centre of Excellence of European Branch of International Federation for Surgery of Obesity, Hazrat_e Rasool Hospital, Tehran, Iran; 3 Urmia University of Medical Sciences, Urmia, Iran; 4 Minimally Invasive Surgery Research Center, Iran University of Medical Sciences, Tehran, Iran

**Keywords:** Bariatric Surgery, Metabolic Surgery, Obesity, Low BMI

## Abstract

**Background:**

Patients with class I obesity may need metabolic and bariatric surgery (MBS) in the presence of obesity-associated medical problems, but MBS in this class of obesity is under debate. This study aimed to investigate the efficacy and safety of MBS in patients with class I obesity.

**Methods and materials:**

This study was a historical cohort carried out on 112 patients with class I obesity with body mass index (BMI) of 30–35 kg/m^2^ with a 24-month follow-up underwent MBS at Rasoul-e-Akram Hospital. The required data were extracted through the Iran National Obesity Surgery Database. The data required for the study consisted of demographic information such as age, gender, and obesity-associated medical problems like type-2 diabetes mellitus (T2DM), hypertension, obstructive sleep apnea, and dyslipidemia before surgery, 6, 12, and 24 months after surgery.

**Results:**

Mean age of the patients was 38.10 ± 10.04 years; mean BMI was 32.96 ± 1.35 kg/m^2^ and 83.9% (n = 94) of patients were female. Out of 18 patients with T2DM, 11 patients (61.11%) had complete remission and seven patients (38.88%) had partial remission. Obstructive sleep apnea, hypertension, dyslipidemia, and gastroesophageal reflux disease were observed in 18 (16.07%), 23 (20.53%), 43 (38.39%), and 13 patients (11.60%) before surgery and resolved at 24-month follow-up. Post-operative complications during the 24-month follow-up were checked to assess safety and there were no De novo gastroesophageal reflux disease, intolerance, leakage, pulmonary thromboembolism, deep vein thrombosis, incisional hernia, hypoalbuminemia (Albumin < 3.5 g/dl), excessive weight loss (BMI < 18.5 kg/m^2^) at any time during 24-months follow-ups and mortality. Early complications occurred as splenic injury in one case (0.89%), wound infection in one patient (0.89%), and extra-luminal bleeding in 10 (8.92%) after surgery, without any mortality.

**Conclusion:**

MBS is safe and effective in class I obesity and can be considered in selected patients with obesity-associated medical problems.

## Introduction

It is estimated that more than 650 million people are obese worldwide [1]. Obesity as a global epidemic is associated with significant health, social, and economic consequences [2]. The association between obesity and psychological/psychiatric problems as well as many chronic diseases such as cardiovascular diseases, type 2 diabetes mellitus (T2DM), hypertension (HTN), physical disability and respiratory diseases has been confirmed [3]. Class I obesity, defined as having a body mass index (BMI) of 30 to 35 kg/m2, has been shown to be associated with a significant increased risk of developing diseases like type 2 diabetes, hypertension, dyslipidemia, obstructive sleep apnea (OSA), and mortality [4]. Extensive evidence supports the significant superiority of metabolic and bariatric surgery (MBS) over non-surgical treatment approaches in patients with a body mass index (BMI) of 27 or higher who are diagnosed with T2DM [5, 6]. Few studies have demonstrated the safety and efficacy of MBS in improving obesity-associated medical problems in class I obesity compared to higher BMI patients [7].

While BMI is a helpful tool for assessing obesity in patients, it is insufficient to classify obesity or determine whether metabolic and bariatric surgery (MBS) is necessary. Other scales, like the Edmonton Obesity Staging System (EOSS), take into account various aspects of obesity, including functional limitations and physical, psychologic, and psychiatric symptoms [8].

Despite new American Society of Metabolic and Bariatric Surgery (ASMBS) and International Federation for the Surgery of Obesity and Metabolic Disorders (IFSO) Indications for MBS [9] that suggested MBS in suitable patients with class I obesity with obesity-associated medical problems that did not have a good respond to non-surgical treatments [10], and some studies confirmed the efficacy of MBS in weight loss and remission of obesity-associated medical problems such as hypertension (HTN), dyslipidemia (DLP) and type 2 diabetes mellitus (T2DM) in patients with Class I obesity [11–13], there are some concerns about MBS safety in this class of obesity.

This study aimed to evaluate the efficacy and safety of MBS in patients with class I obesity and obesity-associated medical problems in a two-year follow-up.

## Methods and materials

### Design and setting

This study was a historical cohort performed on 112 patients with class I obesity and at least one obesity-associated medical problem that did not have a good response to non-surgical therapies and underwent MBS at Rasoul-e-Akram hospital, an International Federation for the Surgery of Obesity and Metabolic Disorders center of excellence **(**IFSO-COE) for MBS. All patients underwent full evaluation by a multi-disciplinary team (MDT) before surgery. The patients were operated by one surgical team with the same techniques and had 24-month follow-up by MDT to evaluate weight loss and remission of obesity-associated medical problems and complications.

### Eligibility criteria

Inclusion criteria were all patients (by census sampling method) with a body mass index of 30 to 35 kg/m^2^, aged between 18 and 75 years who underwent MBS in our center.

### Surgical methods

#### RYGB

Patients were positioned in a slightly reversed Trendelenburg position, under general anesthesia, following the French technique. CO2 insufflation was carried out using a Veress needle, and an optical trocar was inserted in the midline, approximately 10 to 15 cm below the xiphoid process. Two 12-mm trocars were placed as the right and left working hands, while two 5-mm trocars served as an assistant and for liver retraction. A gastric pouch measuring six centimeters in length was constructed, utilizing a 36-French bougie. The gastrojejunostomy was created 100 cm from the Treitz ligament, and a 75 cm Roux limb was formed. The jejunojejunostomy was performed using a 30 mm white endo-stapler, closing both the Petersen and jejunojejunal defects with prolene 2 − 0 sutures.

#### SG

Following the initial steps mentioned previously, which included releasing the angle of His and performing gastrolysis, a sleeve gastrectomy (SG) was performed. The SG was carried out with purple linear endo-staplers, starting 4 cm proximal to the pylorus, over a 36-French bougie. Additionally, an omentopexy was performed along the entire length of the staple line.

#### OAGB

Similar to the initial steps described earlier, a narrow pouch was created distal to the Crow’s foot, using a 36-French tube. A side-to-side gastrojejunostomy was then performed, utilizing a linear 30-mm stapler, approximately 150 cm after the Treitz ligament.

#### Gastric plication

Gastrolysis was initiated 4 cm proximal to the pylorus, employing hemostatic devices, and continued until reaching the His angle about 2 cm from the esophago-gastric junction. Plication was performed in two rows, following a 36-French bougie as a guide.

### Data collection

The required data were extracted through the Iran National Obesity Surgery Database (INOSD) [14] provided by a minimally invasive surgery research center. The data required for the study consisted of demographic information such as age and gender, obesity-associated medical problems like T2DM, HTN, OSA, gastroesophageal reflux disease (GERD), dyslipidemia and hypoalbuminemia before surgery, 6, 12, and 24-month after surgery extracted from the database.

### Outcome definitions

Efficacy was defined as a remission/improvement of obesity-associated medical problems and weight loss outcomes such as total weight loss percent (%TWL) and Excess weight loss percent (%EWL). Safety was checked by assessing early complications such as leak, bleeding, and mortality and late complications such as hypoalbuminemia (Alb < 3.5 g/dl), de novo GERD, and excessive weight loss (BMI < 18.5 kg/m^2^) at any time during 24 months follow-ups [15].

The outcomes of associated medical problems (resolution/improvement) were defined based on “Standardized Outcomes Reporting In Metabolic And Bariatric Surgery“ [16].

T2DM (complete remission: HbA1c < 6%, FBG < 100 mg/dl in the absence of anti-diabetic medications; improvement: reduction in HbA1c and FBG (not meeting the criteria for remission) or decrease in anti-diabetic medication requirement [16].

### Ethical issues

The research adhered to the fundamentals of the Declaration of Helsinki. The Ethical Committee of the Iran University of Medical Sciences approved the protocol for this study (#IR.IUMS.REC.1400.361).

### Statistical analysis

To analyze the data, number and percentage indices were employed for qualitative variables, and quantitative variables mean, standard deviation, mean, and minimum-maximum value indices were utilized. Due to the normal distribution of the data, parametric tests were used such as repeated measure ANOVA, one-way ANOVA, and McNemar test for checking the binary status of obesity-associated medical problems. To check the significance of changes between groups (One anastomosis gastric bypass, Roux-en Y gastric bypass, sleeve gastrectomy, and gastric plication), one-way ANOVA was used for %TWL and %EWL indices at 6, 12, and 24-month follow up in which these indices was significantly different for %EWL at 6-month follow up. Efficacy also was checked by assessing remission of OSA, HTN, dyslipidemia, and T2DM. All descriptive tables and statistical tests were prepared using SPSS software version 21 and the statistical significance level was considered 0.05.

## Results

**Fig. 1:**
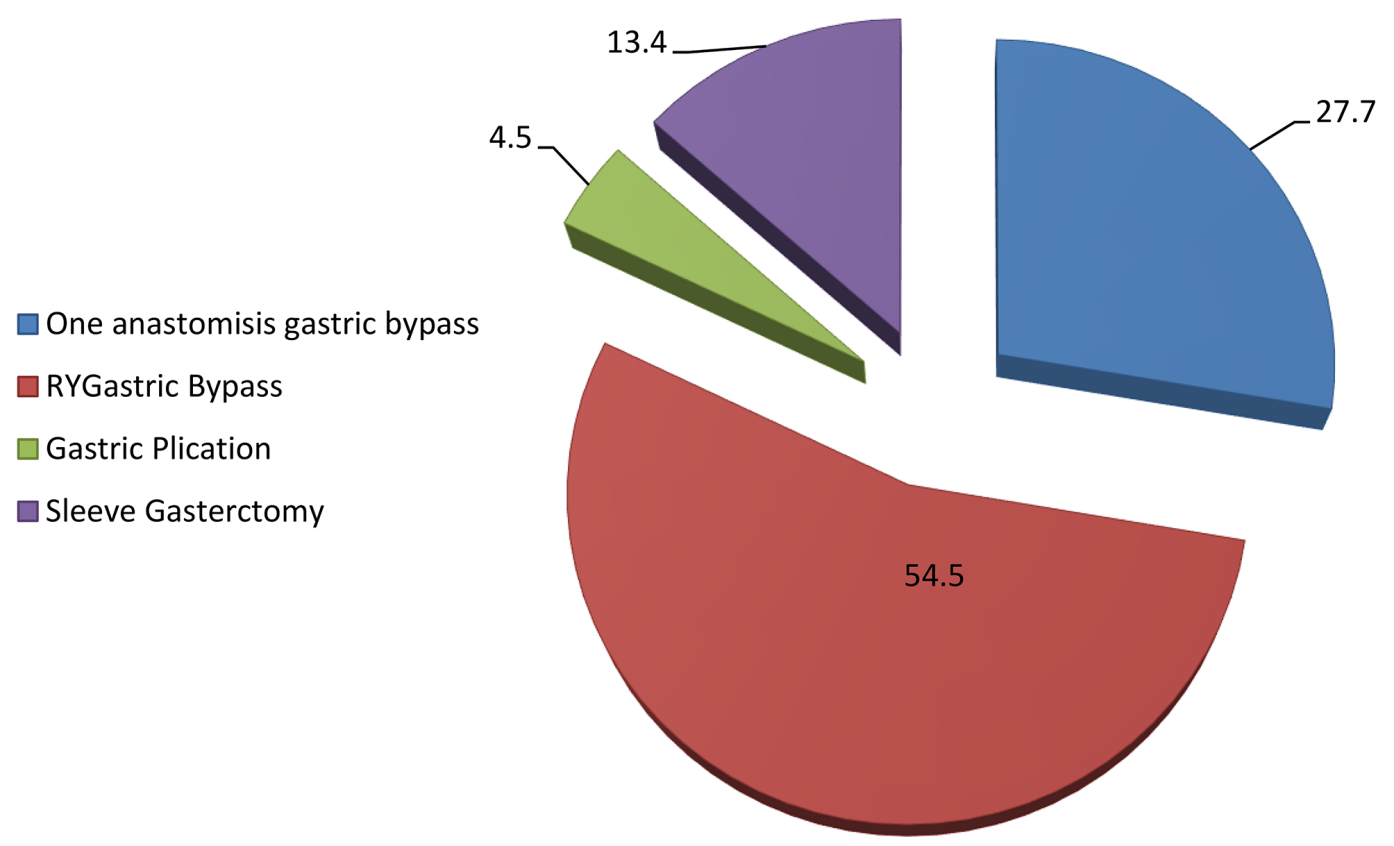
Type of surgeries done on patients with grade I obesity

The mean age of the patients was 38.10 ± 10.04 years; the mean BMI was 32.96 ± 1.35 kg/m^2^ and 83.9% (n = 94) of patients were female (Table 1). The most common type of surgery was Roux-en-Y gastric bypass (RYGB) that performed on 61 patients. The type of MBS is shown in Fig. 1.

**Table 1:** Baseline data of low BMI patients

Variable	Value
Age, Mean ± SD (range),year	38.10 ± 10.04 (14.60–68.60)
Female sex, no. (%)	94 (83.9)
BMI, Mean ± SD (range), kg/m^2^	32.96 ± 1.35 (30.19–34.96)
T2DM, no. (%)	18 (16.07)
OSA, no. (%)	18 (16.07)
GERD, no. (%)	13 (11.60)
HTN, no. (%)	23 (20.53)
DLP, no. (%)	43 (38.39)

BMI: body mass index, BS: before surgery, SD: standard deviation, T2DM: type 2 diabetes mellitus, OSA: Obstructive sleep apnea, GERD: Gastroesophageal reflux disease, HTN: hypertension, DLP: Dyslipidemia

**Fig. 2:**
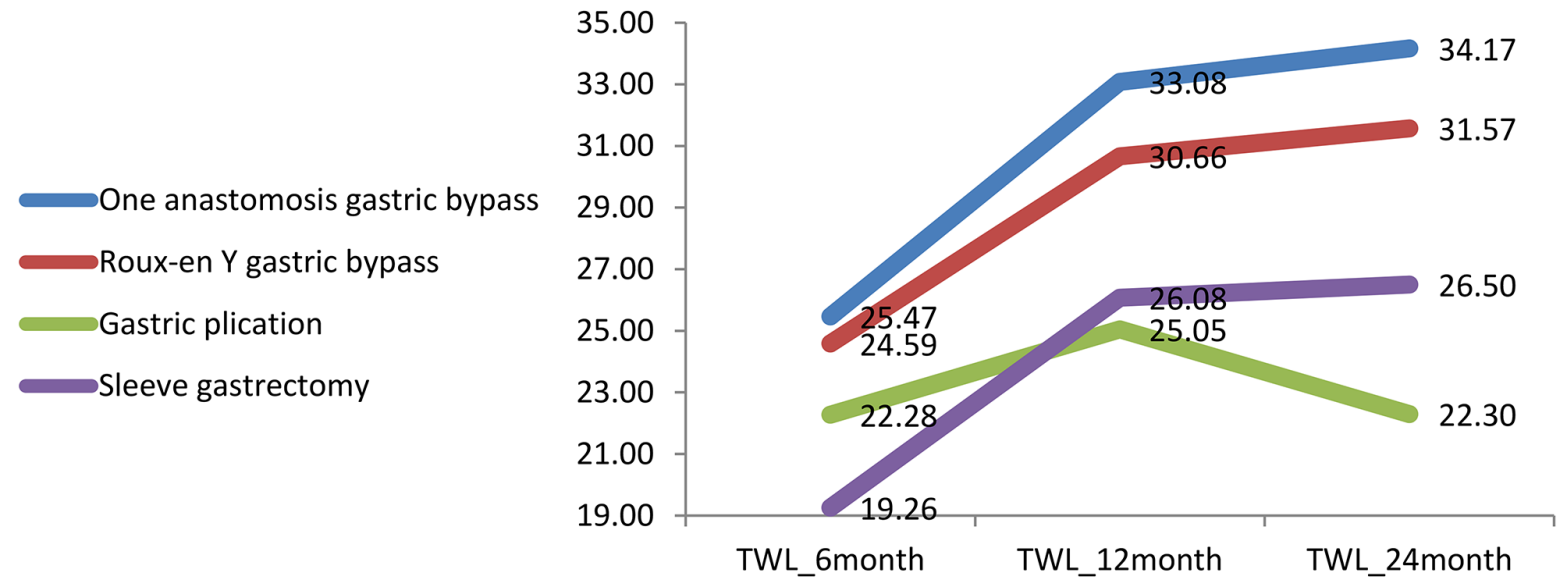
Change of TWL% in three point (6-month, 12-month and 24-month)

The type of MBS was not an effective factor for %TWL or %EWL changes (Table 2). %TWL trend is shown in Fig. 2. Out of 18 patients with T2DM, 11 patients (61.11%) had complete remission (HbA1C less than 6 and FBS less than 100) and seven patients (38.88%) had partial remission (HbA1C less than 6.5 and FBS less than 125). OSA was observed in 18 patients (16.07%) before surgery and reached zero at 24-month follow-up. HTN was observed in 23 patients (20.53%) before surgery and reached zero at 24-month follow-up. Dyslipidemia was observed in 43 patients (38.39%) before surgery and reached zero at 24-month follow-up. Also, GERD was observed in 13 patients (11.60%) before surgery and reached zero at 24-month follow-up (Table 3).

**Table 2:** %TWL and %EWL changes by the type of surgery at 6, 12 and 24-month follow up using one-way ANOVA test

Variable	OAGB	RYGB	Gastric plication	SG	***P***-value
**%TWL**					
6 month	26.88 ± 9.88	23.72 ± 7.62	23.16 ± 11.78	24.18 ± 9.29	0.498
12 month	33.70 ± 10.09	29.86 ± 8.98	25.04 ± 11.77	28.94 ± 11.27	0.289
24 month	33.58 ± 10.96	31.59 ± 7.86	22.29 ± 11.35	26.50 ± 12.41	0.156
**% EWL**					
6 month	70.70 ± 25.58	58.27 ± 23.30	65.48 ± 22.85	85.00 ± 42.60	0.023
12 month	83.75 ± 22.72	72.48 ± 21.69	70.57 ± 26.17	94.29 ± 46.64	0.068
24 month	87.46 ± 39.20	74.83 ± 18.73	62.53 ± 26.82	75.41 ± 49.23	0.261

OAGB: One anastomosis gastric bypass; RYGB: Roux-en Y gastric bypass; SG: Sleeve gastrectomy

**Table 3:** Efficacy (complete remission) following bariatric surgery during 24-month follows up

Comorbidities	Before surgery no. (%)	After surgery no. (%)	Sig.
OSA	18 (16.07)	0 (0)	< 0.001
HTN	23 (20.53)	0 (0)	< 0.001
DLP	43 (38.39)	0 (0)	< 0.001
T2DM	18 (16.07)	7 (6.25)	< 0.001
GERD	13 (11.60)	0 (0)	< 0.001

OSA: Obstructive Sleep Apnea, HTN: Hypertension; DLP: Dyslipidemia; Type 2 Diabetes Mellitus, T2DM; GERD: Gastroesophageal Reflux disease

Post-operative complications during the 24-month follow-up were checked to assess the safety as reported based on the Clavien-Dindo classification in Table 4.

**Table 4:** Post-operative complications during 24-month follows up based on clavien-dindo classification

Complications			Value
	Minor	Bleeding	9 (8%)
		DVT	0
		Wound Infection	1 (0.8%)
		Total	10 (8.8%)
	Major	Bleeding needs transfusion (Splenic Injury)	1 (0.8%)
		Leak	0
		Total	1 (0.8%)
Late	Minor	GERD	0
		Anemia	4 (3.5%)
		Hypoalbuminemia	0
		Excessive Weight Loss	0
		Total	4 (3.5%)
	Major	Intractable GERD	0
		Internal Hernia	0
		Upper GI Bleeding	0
		Severe resistant anemia	0
		Total	0

## Discussion

The findings of this study provide compelling evidence supporting the effectiveness and safety of metabolic and bariatric surgery (MBS) in patients with Class I obesity, as demonstrated in two years of follow-up. Regardless of the specific type of MBS procedure performed, our patients experienced no instances of mortality and did not exhibit excessive weight loss.

According to the updated guidelines from the 2022 American Society of Metabolic and Bariatric Surgery (ASMBS) and International Federation for the Surgery of Obesity and Metabolic Disorders (IFSO) [9], the indications for metabolic and bariatric surgery have changed from the previous NIH criteria [17]. According to the new guidelines, individuals with a BMI of 30-34.9 kg/m2, who have metabolic disease and have not achieved significant or long-lasting weight loss or improvement in co-morbidities through non-surgical methods, should be considered for MBS.

Class I obesity is associated with an increased risk of various conditions, including T2DM, HTN, DLP, metabolic syndrome, non-alcoholic fatty liver disease (NAFLD), and obesity-related cancers. However, relying solely on BMI as a criterion for MBS may not provide a complete picture, as BMI alone does not accurately reflect the individual’s underlying biological factors [18].

To address this limitation, alternative classification tools such as the Edmonton Obesity Staging System (EOSS) take into account multiple aspects of obesity, including physical symptoms, psychological/psychiatric symptoms, and functional limitations. These comprehensive tools offer a more accurate prediction of total obesity-related mortality and can effectively identify patients who are genuine candidates for MBS. Therefore, considering additional factors beyond BMI is crucial in determining the appropriate candidates for MBS [18].

On the other hand, the suggestion of MBS only according to BMI may not be completely true, because BMI alone cannot be a perfect predictor of patients’ body biological components [18].

So, other classification tools such as the Edmonton Obesity Staging System (EOSS) which includes all aspects of obesity such as physical, psychologic/psychiatric symptoms, and functional limitations are more accurate in predicting total obesity-related mortality and can be useful for determining which patients are true candidates for MBS [8]. According to a recently published global survey, there are different MBS approaches in the treatment of patients with low BMI around the world [19].

The most important issue is the safety of MBS in these patients which should be evaluated in different studies. In a study conducted by Jackson et al., the Metabolic and Bariatric Surgery Accreditation and Quality Improvement Program (MBSAQIP) database was evaluated to assess the safety of MBS in patients with class I obesity. The findings of the study confirmed the short-term safety of MBS in this specific patient population. The analysis provided evidence supporting the favorable safety profile of MBS procedures for individuals with class 1 obesity [7].

In this study, we did not find any significant morbidity after MBS. We did not have any hypoalbuminemia, but anemia occurred in 4 (3.57%) of the patients who were all treated with Iron supplement prescription. Maiz et al., reported anemia in 0.5% and 1.8% of patients with class I obesity who underwent sleeve gastrectomy (SG) and RYGB respectively at one-year follow-up [20]. They also reported 1.1% portomesenteric vein thrombosis in 1.1% of patients who underwent SG and 0.2 and 0.4% leak after SG and RYGB respectively [20].

In a study conducted by Verban et al., [11] the outcomes of patients who underwent primary SG were compared between those with a BMI below 35 kg/m2 and those with a BMI equal to or above 35 kg/m2. The results revealed that patients with lower BMI experienced higher resolution rates for T2DM, HTN, and DLP. Additionally, they were more likely to achieve a normal weight following SG.

Studer et al. reported the 5-year outcomes of RYGB and SG in a group of 37 patients with class I obesity. They found that remission of HTN was achieved in 42% of the patients, and remission of DLP was observed in 64% of the cases. They also noted a 12% incidence of de novo reflux after SG, with no excessive weight loss or mortality observed [12].

Kular et al. reported anemia (3.9%), hypoalbuminemia (0.8%), excessive weight loss (2.3%), and bile reflux (0.8%) in patients with class I obesity who underwent one anastomosis gastric bypass (OAGB) in a 7-year follow-up which all treated by conservative management [21]. Hong et al. and Cevallos et al. also confirmed the safety of SG in this class of obesity [22, 23].

According to a study conducted by Gamme et al., there was no statistically significant difference in the risk of postoperative complications between individuals with class I obesity and those with Class II and higher obesity after SG and RYGB. The study’s findings indicate that patients in Class I obesity do not face a higher risk of postoperative complications compared to individuals with higher levels of obesity [24].

This study similar to previous studies confirms the safety of MBS in patients with class I obesity.

Although BMI alone cannot predict the T2DM outcomes after medical or surgical treatment modalities [25], it has been demonstrated that MBS has a more significant and durable effect on remission and improvement of T2DM compared to non-surgical treatments such as GLP-1 analog, SGLT-2 inhibitors in patients with BMI < 35 Kg/m^2^ [26, 27].

According to our study, 11 out of 18 patients (61.1%) achieved complete remission and seven (38.9%) achieved partial remission after undergoing MBS, with a two-year follow-up.

In a study by Cevallos et al., 73.8%, 52.2%, and 50% of patients saw complete remission of T2DM after 24, 36, and 60 months, respectively [23]. Berry et al. reported complete and partial remission in 60% and 40% of patients, respectively, after a three-year follow-up after SG [28]. Baldwin et al. reported 100% remission in T2DM at 12 months after SG and RYGB in Patients with class I obesity [29].

The current study showed complete remission of OSA, GERD, DLP, and HTN in 100% of patients in our 2-year follow which confirms the efficacy of MBS in remission of obesity-associated medical problems in patients with class I obesity. These excellent results are reported in similar previous studies with different metabolic/bariatric surgical procedures [20, 28–31].

Our study also found that the weight loss outcomes after OAGB, RYGB, gastric plication, and SG were 33.58%, 31.59%, 22.29%, 26.50% in terms of total weight loss percentage, and 87.46%, 74.83%, 62.53%, and 75.41% in terms of excess weight loss percentage (EWL%), respectively. These results are consistent with previous studies and further support the efficacy of MBS in weight loss for patients with class I obesity. It is important to note that MBS should only be considered after non-surgical treatments have failed for patients with class I obesity and related diseases [20, 22, 23, 28–30, 32] that confirm the efficacy of different types of MBS in weight loss outcomes in patients with class I obesity.

In patients with class I obesity, MBS should be considered after the failure of non-surgical treatments such as medical therapies and lifestyle modification in the treatment of obesity and weight-related diseases [10].

### Limitations of the study

Our study represents an acceptable sample size with a year’s follow to evaluate class I obesity patients who have undergone MBS. Also, we assessed the effects of different types of MBS on obesity-related diseases. However, we acknowledge several limitations. The study was retrospective with the limitations of this design. Because of this, we had to deal with a lack of information and insufficient follow-up resulting in low cases. Additionally, MBS was performed by different surgeons, and surgical technique details for example biliopancreatic limb length were not included in this study.

Hopefully, further larger trials with longer follow-ups would generate the necessary evidence to allow a better patient selection for MBS based on their associated conditions and individual considerations in low BMI patients.

## Conclusion

Class I obesity is accompanied by many obesity-associated medical problems that treated in the early stages could avoid becoming more severe. Our study showed that MBS is a safe and effective option that allows patients to reach a successful weight loss with considerable obesity-associated medical problems remission and a low rate of complications. Further studies are needed to investigate the results of MBS in patients with low BMI in the long run.

## Data Availability

The datasets used during the current study are available from the the corresponding author on reasonable request.
